# A mechanism-based classification of pain in multiple sclerosis

**DOI:** 10.1007/s00415-012-6579-2

**Published:** 2012-07-04

**Authors:** A. Truini, P. Barbanti, C. Pozzilli, G. Cruccu

**Affiliations:** 1 Department of Neurology and Psychiatry, Sapienza University, Viale Università 30, 00185 Rome, Italy; 2 Don Gnocchi Foundation, Milan, Italy; 3 San Raffaele IRCCS, Rome, Italy; 4 MS Center, S. Andrea Hospital, Rome, Italy

**Keywords:** Multiple sclerosis, Pain prevalence, Treatment trial, Neuropathic pain, Spasticity, Migraine

## Abstract

Pharmacological treatment of pain in multiple sclerosis (MS) is challenging due to the many underlying pathophysiological mechanisms. Few controlled trials show adequate pain control in this population. Emerging evidence suggests that pain might be more effectively classified and treated according to symptoms and underlying mechanisms. The new mechanism-based classification we propose here distinguishes nine types of MS-related pain: trigeminal neuralgia and Lhermitte’s phenomenon (paroxysmal neuropathic pain due to ectopic impulse generation along primary afferents), ongoing extremity pain (deafferentation pain secondary to lesion in the spino-thalamo-cortical pathways), painful tonic spasms and spasticity pain (mixed pains secondary to lesions in the central motor pathways but mediated by muscle nociceptors), pain associated with optic neuritis (nerve trunk pain originating from *nervi nervorum*), musculoskeletal pains (nociceptive pain arising from postural abnormalities secondary to motor disorders), migraine (nociceptive pain favored by predisposing factors or secondary to midbrain lesions), and treatment-induced pains. Identification of various types of MS-related pain will allow appropriate targeted pharmacological treatment and improve clinical practice.

## Introduction

Research into pain and pain management has long debated the relationship symptom-mechanism-treatment. Newly proposed screening questionnaires, and diagnostic procedures such as quantitative sensory testing, pain-related evoked potentials, and skin biopsy [19, 33], have advanced the mechanistic approach to pain management leading to the development of the so-called sensory profiles [6, 9, 20, 59].

Making a specific diagnosis, understanding the underlying pathophysiological mechanism and implementing the proper treatment strategy depends first of all on clearly defining the various types of pain.

In this partly systematic but largely argumentative review, we sought epidemiological and pharmacological studies on MS-related pain. Seeking support for our proposed mechanism-based classification for MS-related pain, we then reviewed studies that help to define and understand the various types of MS-related pain. From current knowledge, our own clinical experience, and information gained from our review, we then tried to classify the different types of MS-related pain according to the underlying pathophysiological mechanism.

We distinguish five pain categories, nociceptive, neuropathic, psychogenic, idiopathic, and mixed. These categories are variably established in the literature. A PubMed search including papers published from inception date to 2011 showed how many articles used these terms:

- neuropathic pain: 7,759,
- nociceptive pain (378) or inflammatory pain (1,868); total: 2,246,
- psychogenic pain (171) or somatoform pain (136) or pain behavior (244); total: 551,
- idiopathic pain: 74,
- mixed pain: 46.

To sharpen the distinction between nociceptive pain (whether inflammatory or non-inflammatory), namely pain resulting from nociceptor activation by true or potentially tissue-damaging stimuli, and neuropathic pain, the International Association for the Study of Pain (IASP) has redefined neuropathic pain as pain arising directly from a lesion or disease affecting the somatosensory system [105]. The previous definition (pain initiated or caused by a primary nervous system lesion or dysfunction) left room for numerous conditions that are neither really or wholly neuropathic. For example, the word *dysfunction* allowed inclusion of pain related to secondary functional and neuroplastic changes in the nervous system resulting from sufficiently strong nociceptive stimulation, e.g., central sensitization [14, 106]. The phrasing *initiated* by a primary lesion in *the nervous system* left room for any pain in neurological disease, in particular all musculoskeletal pains secondary to movement disorders [26, 73, 91], a major problem in patients with MS.

Among the five categories of pain, one that is difficult to define is psychogenic pain. This term refers to both primary psychiatric conditions such us somatoform pains associated with anxiety and depression [30, 36, 47, 53, 84], and also to the superimposed psychogenic components that often develop in patients with chronic refractory pain. This possibility is important to remember because patients with chronic pain may develop a psychogenic component that overwhelms the original organic disease and lose compliance to the theoretically correct therapy (a condition also called *pain behavior*) [99, 108].

The category idiopathic pain encompasses several poorly understood or controversially interpreted chronic pain conditions, including fibromyalgia, irritable bowel syndrome, interstitial cystitis, and persistent idiopathic facial pain [24, 93], all of which may share a common genetic predisposition or be related to some kind of brain dysfunction [51, 67, 96]. Because countless patients experience idiopathic pain, the field attracts major research efforts.

The term mixed pain is the least used and the concept remains controversial [8, 87]. Indeed, many pain clinicians and investigators deny its usefulness, arguing that the term “mixed” adds nothing to “coexisting”. Because many patients may experience more than one type of pain and may have two or more diseases, we need to clarify the difference between “coexisting” and “mixed”.

Coexisting pains are unrelated: their causes and pathophysiological mechanisms differ and they require independent treatment. For instance, in a patient with syringomyelia involving the C5 dermatome who also has a periarthritis shoulder, two independent conditions that just happen to share a similar territory exist and must be independently managed. A more intriguing example comes from a patient with trigeminal neuralgia affecting the mandibular division who has a coexisting temporomandibular disorder. This patient may report the typical electric shock-like attacks of trigeminal neuralgia and a dull, deep, and ongoing pain in the same territory and must therefore be distinguished from a patient with atypical trigeminal neuralgia, a neuropathic pain that entails both paroxysmal and background pain [70]. Neither patient has mixed pain.

Conversely, in mixed pain, the *same* disease causes different pains through different pathophysiological mechanisms that are often difficult to separate and quantify and may therefore raise management problems. A frequent condition that fits this definition is low back pain with sciatica, including both nociceptive components arising from muscles, ligaments, and joints, and neuropathic component arising from the spinal root. When the involved spinal roots innervate proximal territories the neuropathic and nociceptive components may be difficult to separate. Besides spinal root compression, inflammatory mediators originating from the degenerative disc may induce radicular pain without any mechanical compression or nociceptive sprouts within the degenerated disc may give rise to local neuropathic pain [27]. An important example of mixed pain comes from cancer pain. When lung cancer invades the brachial plexus and the patient feels pain projected to the hand, neuropathic pain clearly adds to nociceptive pain. Emerging evidence, however, suggests that cancer is bound to produce mixed pain with a less obvious mechanism and less clear symptoms: the tumor invading the surrounding tissues destroys the local nerve endings thus inducing regenerating sprouts that are rich in a variety of pain-related channels and also induce central sensitization; so far, this has been well established for bone cancer [49, 62, 76, 117]. In this case, it is impossible to distinguish between and quantify the nociceptive and neuropathic components.

For multiple sclerosis, the concept of mixed pain is especially important because two types of MS-related pain should be considered mixed: tonic painful spasms and spasticity pain (see below).

## Pain in multiple sclerosis: inadequacy of epidemiological studies

Pain is a major burden for patients with MS [4, 71, 81]. The estimated prevalence of MS-related pain ranges widely from 26 to 86 % [69, 71]. The high variability reflects differences in the criteria used to define the various types of MS-related pain, the types of pain assessed in the epidemiological survey, the study sample (e.g., population cohorts, hospitalized patients), and the research methods (mail surveys, administrative database queries, and in-person history and examination) [71]. In a systematic review of pain related to MS, O’Connor and colleagues [71] found that most studies reported a prevalence higher than 50 %. In a meta-analysis (restricted to studies that provided sufficient information and sufficient quality of methodology), they calculated that 633/854 (74 %) outpatients had pain within 1 month [71].

The discrepancy in study design and methods accounts also for differences in the reported risk factors for the development of pain related to MS (patient’s age, duration of disease, disease course, and disability). Whereas some studies reported an association between one or more of these factors and pain [34, 97], others did not [10, 44, 73]. A large sample study on the prevalence of pain in MS [97] identified as the main factors associated with the development of pain a longer duration of disease, older age, a non-relapsing-remitting MS course and greater disability. A possible drawback of this finding is that all these factors are intermingled and the study lacked a multivariate analysis to distinguish the role of single factors. The role of the various risk factors in the development of pain therefore awaits clearer answers.

## Pain in multiple sclerosis: insufficiency of pharmacological trials

A search of the electronic literature from date of inception to April 2012 showed that only 12 randomized placebo-controlled trials (RCTs) have assessed pharmacological—non disease-modifying—treatment of pain in MS (see “Appendix” for details about this search). For comparison with MS, we chose diabetic painful neuropathy, because the prevalence of pain in diabetes has been reliably assessed (most studies agree on values between 16 and 19 %) [22, 23] and is far lower than the estimated prevalence of pain in MS (74 %). Nevertheless, when we surveyed the literature, we found 188 RCTs assessing pain in diabetic neuropathy but only 12 assessing pain in MS. Articles on diabetes are arguably far more numerous than those on MS and drug companies tend to prefer a huge market such as that provided by diabetes. Another reason, however, for the dearth of drug trials is that until a few years ago, most physicians tended to underestimate the importance of pain in MS [71, 103, 107]. Indeed, when we compared RCTs investigating spasticity and pain in MS, we found that a considerably larger number assessed spasticity than pain (33 vs. 12) and several of the latter were RCTs targeting spasticity that also included pain relief as one of the outcome variables (Table 1).

**Table 1:**
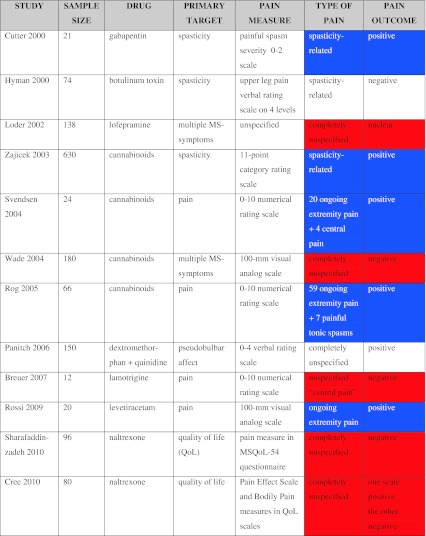
Double-blind placebo-controlled RCTs for pain treatment in multiple sclerosis

Blue cells highlight studies that specified the type of pain and had an unambiguously positive outcome on pain. Red cells highlight studies that did not specify the type of pain and did not have an unambiguously positive outcome on pain. Note that the accuracy in specifying the type of pain and outcome was uncoupled in only two of 12 trials [39, 75]

Besides the scarcity of RCTs assessing pain related to MS, our review identified other problems. Of the 12 RCTs reviewed, we found only four dedicated to pain assessment [13, 86, 88], whereas all the others included some pain measure as an item in quality-of-life scales or spasticity scales, and even more importantly, five studies failed to describe the type of pain [15, 54, 75, 94, 113], and only one generically mentioned “central pain”, without specifying further or distinguishing paroxysmal pain (such as trigeminal neuralgia) from ongoing pain (such as extremity pain) [13]. Of these six trials, only one, testing dextromethorphan/quinidine, was unambiguously successful in relieving pain [75].

One study investigated ongoing extremity pain [88], one ongoing extremity pain and a few patients with painful tonic spasms [86], one central pain, but specifying that most patients had extremity pain [100], and three others assessed pain related to spasticity and spasms [21, 39, 116]. Of these six trials, only one, using botulinum toxin injections, was unsuccessful in relieving pain [39]. Although the success of a trial depends strongly on the active treatment, among all the trials testing cannabinoids, the only one reporting no significant pain relief was the one that failed to take into account the type of pain [113]. Our finding that the frequency of success differs significantly (*p* = 0.02; Chi-square) between studies that adequately categorized the type of pain and those that did not (Table 1) confirms that pain mechanisms do differ and pharmacological trials should aim to target specific types of pain.

Finally, whereas many trials assessed cannabis derivates, no RCT exists on the drugs that have for long been most popular in treating neuropathic pain (amitriptyline and carbamazepine) or are currently popular (pregabalin and serotonin noradrenaline reuptake inhibitors) [7, 102].

## Proposed classification for pain in multiple sclerosis

In 2008, O’Connor and colleagues [71] published a useful review that also proposed a classification for pain in multiple sclerosis, as we are doing. With respect to theirs, our classification is more based on pathophysiological mechanisms and response to treatment, and most notably differs in our introduction of the mixed pain category.

### Neuropathic pains

Considering the two most reliable studies that assessed the prevalence of neuropathic pain through clinical examination, 129/414 patients (31 %) had one or more types of neuropathic pain [73, 101] (Table 2).

**Table 2:** Proposed classification of pain in multiple sclerosis

Types of pain (% frequency)	Possible mechanisms	Theoretical treatment
*Neuropathic pains*		
1. Ongoing extremity pain (12–28 %)	Thalamic or cortical deafferentation pain by multiple lesions along the spino-thalamo-cortical pathways	Antidepressants Cannabinoids
2. Trigeminal neuralgia (2–5 %)	High-frequency discharges ectopically generated by intra-axial inflammatory demyelination plus extra-axial mechanical demyelination of the trigeminal primary afferents	1. Sodium-channel blockers 2. Microvascular decompression
3. Lhermitte’s phenomenon (15 %)	High-frequency discharges ectopically generated by demyelination of the dorsal column primary afferents	Sodium-channel blockers
*Mixed pains*		
1. Painful tonic spasms (6-11 %)	High-frequency discharges ectopically generated by demyelination in the corticospinal pathways induce spasmodic muscle contractions, which in turn induce ischemic muscle pain	Sodium-channel blockers Cannabinoids
2. Spasticity pain (<50 %)	Disinhibition by a corticospinal tract lesion enhances the tonic stretch reflex, which in turn gives rise to excessive muscular work and mechanical muscle pain	Antispastic agents Cannabinoids
*Nociceptive pains*		
1. Nerve trunk pain associated with optic neuritis (8 %)	Endoneural inflammation activates intraneural nociceptors of the nervi nervorum	Corticosteroids
2a. Musculoskeletal pains induced by postural anomalies (?)	Postural anomalies secondary to motor disturbances	Standard pharmacological treatment and physiotherapy for mechanical musculoskeletal pain
2b. Back pain (10–16 %)	No evidence has yet excluded the possibility that in addition to the aforementioned postural anomalies MS may itself directly contribute to back pain	Standard pharmacological treatment and physiotherapy for back pain
3a. Migraine (34 %)	The two diseases share predisposing factors Midbrain lesions	Standard treatment for migraine
3b. Tension-type headache (21 %)	No evidence against two chance coexisting conditions	Standard treatment for tension headache
4. Treatment-induced pains (?)	See text	
*Other pains*	See text	

The % frequencies calculated in the first column take into account the total number of MS patients. Because many patients may have two or more types of pain, the sum of these % frequencies exceeds 100. The most reliable estimate for total pain prevalence in MS is 74 %

<50 %: Because pain is frequently though not invariably manifested by patients with spasticity its prevalence must be lower than that of spasticity, which is about 50 %

(?): we have no direct estimate; the estimates of other types of pain cannot be simply subtracted from estimates of pain in general because many patients have more than one type of pain

In the third column, cannabinoids are always in second row because their mechanism is less specific (not because second-line treatment)

#### Ongoing extremity pain

Although most published studies on MS describe this type of pain as *dysesthetic extremity pain*, this terminology contrasts with the definition of sensory disturbances proposed by the IASP and generally accepted in pain research: dysesthesia indicates an “unpleasant abnormal sensation, whether spontaneous or evoked” [40]. To avoid confusion, we therefore recommend defining this condition, characterized by constant (and often burning) pain that predominantly affects legs and feet, as *ongoing extremity pain* [107]. The reported lifetime prevalence rates range between 12 and 28 % [69, 71]. This type of pain is more common in patients with the primary progressive or the progressive-relapsing types of MS, and lowest in the relapsing–remitting type [11, 69]. Patients with this type of pain tend on average to be more disabled than those without pain [69].

**Fig. 1:**
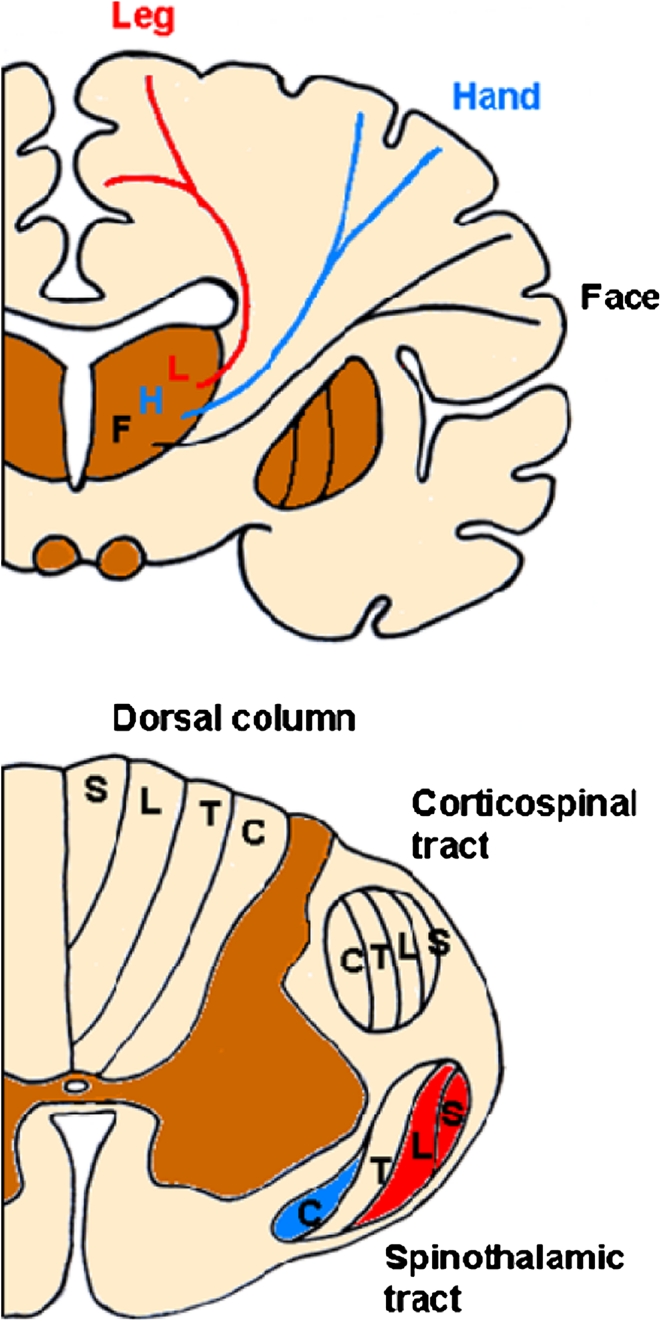
Somatotopy of the spinothalamocortical system. Schematic drawing of a coronal section of the brain (*above*) and an axial section of the spinal cord at cervical level (*below*). In the brain, F, H, and L refer to face, hand, and leg. In the spinal cord C, T, L, and S refer to cervical, thoracic, lumbar, and sacral. In the spinal cord, unlike the dorsal columns and similarly to the corticospinal tract, the spinothalamic tract keeps the fibres for the lower limb (L and S) more superficial. In more caudal sections, the arrangement is identical to that of the corticospinal tract, i.e., in layers with sacral fibers external and cervical fibers internal. At the cervical level, the spinothalamic fibers tend progressively to assume the medial (cervical)–lateral (sacral) arrangement that will be maintained throughout the brainstem, where the additional trigeminothalamic fibers join the ascending tract medially. In the ventrobasal complex of the thalamus, the arrangement is still medial–lateral with neurons for the face medial and those for the leg lateral, but the sensory homunculus has the opposite somatotopic organization, thus the thalamocortical fibers for the face must diverge laterally, whereas those for the leg ascend vertically, traveling close to the lateral ventricle. In the spinal cord, brainstem, and brain, the spinothalamic fibers for the leg travel close to the CSF

The pathophysiology of ongoing extremity pain is poorly understood. Magnetic resonance imaging studies usually show plaques in the cervical and thoracic spinal cord. Hence, this type of pain may arise from lesions in the spinal cord nociceptive pathways. This pathophysiological view receives support from clinical studies in patients with this type of pain showing that thermal-pain sensitivity, sensitivity mediated by the spinothalamic system, is more likely to be affected than lemniscal sensitivities [74]. The bilateral and relatively distal distribution is more difficult to explain. Ample evidence (including studies using electroneurography and skin biopsy) excludes peripheral nerve involvement [73, 78]. Patients with this type of pain presumably have bilateral central lesions, probably multiple lesions, predominantly affecting the feet and legs because the relevant spinothalamic fibres run a longer course and are somatotopically located closer to the spinal cord surface. Fiber length and a location closer to the cerebrospinal fluid (CSF) [64] both increase the probability of demyelinating processes developing (Fig. 1). Brain white matter lesions might also contribute, because most of them are periventricular [64]. Heading for the primary somatosensory area, the thalamocortical fibers for the face diverge laterally, whereas those for the foot ascend close to the lateral ventricle (Fig. 1). Although the opercular-insular cortical areas undoubtedly intervene in pain processing, ample evidence now shows that also in humans the primary somatosensory area plays an important role [42, 46, 83]. In brief, the distal and bilateral distribution of this kind of MS-specific pain probably depends on the length of the spinal thalamocortical system dedicated to the lower extremity and on its somatotopic location in the spinal cord and brain.

#### Trigeminal neuralgia

Trigeminal neuralgia (TN) consists of paroxysmal attacks of electric-shock-like sensations that may develop spontaneously or be evoked by innocuous stimuli in specific facial or intraoral areas (trigger zones). By definition, typical TN is a pain syndrome that arises without a clinically manifest sensory deficit. TN is termed *classic* when investigation identifies no cause other than a neurovascular contact or *symptomatic* when secondary to major neurological disease [17, 31, 35]. Symptomatic TN is frequently related to MS, patients with MS having a 20-fold increased risk of trigeminal neuralgia [48]. About 2–5 % of patients with MS have typical TN [38, 43, 48, 73, 97, 101].

Multiple sclerosis-related TN has for long been attributed to a demyelinating plaque in the pons, as postmortem specimens indicate [43, 55]. The plaque theory nevertheless contrasts with the frequent neuroimaging finding of a neurovascular contact with the trigeminal root in patients with TN and MS and the existence of some patients with MS who have TN as the sole clinical manifestation; others therefore proposed that in most patients with MS, TN merely reflects the high frequency of neurovascular contacts in the normal population [5, 12, 25]. Indeed, histopathological studies of surgical specimens describe demyelination in the proximal, centrally myelinated part of the trigeminal root in patients with MS-related TN and in those with classic TN [56, 57]. A clear-cut difference, however, is that trigeminal reflex testing is abnormal in 89 % patients with MS-related TN, but in only 3 % of patients with classic TN [17, 31].

**Fig. 2:**
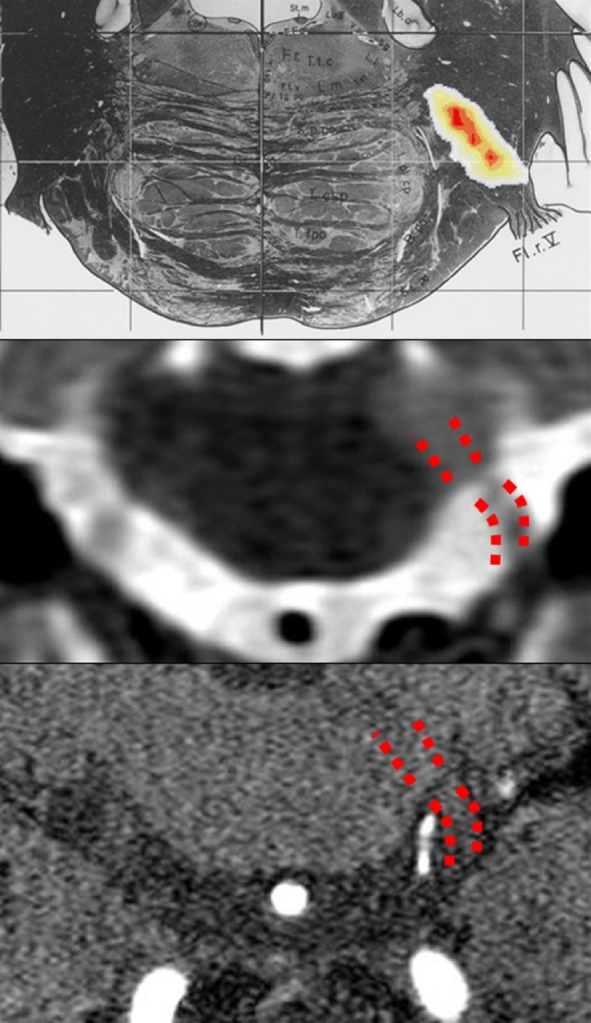
A dual mechanism for trigeminal neuralgia in MS patients.* Top* a voxel-based analysis (see [18] for methods) of the MRI scans in 50 patients with MS and trigeminal neuralgia showed that the area of maximal probability of lesion (*red* = *p* < 0.0001) is located along the intrapontine course of the trigeminal primary afferents [16]. The *colored area* of high probability of lesion is displayed at its proper location on the relevant section of a stereotactic atlas to show the corresponding anatomical structures [92].* Mid* T2-weighted MRI image showing an area of demyelination on the intrapontine course of trigeminal primary afferents in a patient with trigeminal neuralgia associated with MS. In this representative patient, MS was disclosed by the onset of right trigeminal neuralgia at the age of 49 years. *Bottom* MRA image showing a cerebellar artery that distorts the right trigeminal root at its entrance into the pons, in the same subject as above. *Mid and bottom* MRI images have been flipped vertically and horizontally to correspond to the atlas image on *top*. Although in most patients with MS and trigeminal neuralgia, MRI shows a plaque in the area shown in the *top panel*, in several it does not, whereas it often discloses (when looked for) a neurovascular contact. MS patients (regardless of the presence of a plaque on MRI) usually have a typical trigeminal neuralgia indistinguishable from the classic neuralgia attributed to neurovascular contact, but several characteristics differentiate them from both MS patients without trigeminal neuralgia and patients with classic trigeminal neuralgia (see text). We propose that in many patients two mechanisms contribute to the development of trigeminal neuralgia in MS: intra-axial inflammatory demyelination [56] plus extra-axial mechanical demyelination [57] both affecting the same nerve fibers in the root entry zone (*red dots*)

A recent neuroimaging-neurophysiological study in 130 patients with MS, 50 having typical TN, confirmed that in most patients TN was caused by a demyelinating plaque along the intra-axial primary afferents [16]. Other findings in that study nevertheless showed that the TN-group differed from the other patients with MS, both those with trigeminal symptoms other than TN and those with no trigeminal involvement. Only the patients with TN showed the right-left asymmetry typically seen in classic TN and had symptom onset at an older age than MS patients without TN and younger age than patients with classic TN, suggesting that in patients with MS, some other mechanism contributes to the development of TN, probably a neurovascular contact, acknowledged as the most frequent cause of classic TN [17, 31, 35]. These two pathogenetic mechanisms, MS plaque, and a neurovascular contact, would act on the same primary axons and both would produce demyelination [56, 57]. A dual mechanism involving MS and a neurovascular contact (Fig. 2) receives support from neurosurgical studies on the outcome of microvascular decompression because patients with MS-related TN, despite benefiting less than patients with classic TN, experienced considerable pain relief [5, 12, 25].

#### Lhermitte’s phenomenon

Lhermitte’s phenomenon is defined as “a transient short-lasting sensation related to neck movement and felt in the back of the neck, lower back, or in other parts of the body” [2, 71]. Although not unique to MS, Lhermitte’s phenomenon is frequently associated with MS. In many patients, this symptom is transient, and manifests only for some weeks then resolves spontaneously [69]. The reported prevalence of Lhermitte’s phenomenon varies widely. The three studies that analyzed Lhermitte’s phenomenon in at least 100 patients with MS, found it in 313 patients out of 2,085, yielding an overall prevalence of 15 % [2, 45, 97]. Lhermitte’s phenomenon probably depends on a demyelinating plaque in the dorsal columns at cervical level, as suggested by two MRI studies that found a strong association between Lhermitte’s phenomenon and the presence of plaque formations in the posterior cervical spine [2, 32].

Given the quality and duration of pain (patients often describe it as a very short-lasting electric-shock-like sensation), very similar to—though less intense than—the paroxysms of trigeminal neuralgia, we hypothesize the same mechanism, i.e., ectopic generation of a high-frequency discharge along the intra-axial portion of first-order sensory neurons. Were this the responsible mechanism, patients should benefit from sodium-channel blockers. Although Lhermitte’s phenomenon is a frequent sensory disturbance in patients with MS, few patients report this symptom spontaneously and only few consider it as painful. Probably for this reason, only one study directly dealt with treatment of this condition [89]. This controlled study investigated the effect of intravenously injected lidocaine and oral mexiletine in 30 patients with paroxysmal MS-related sensory disturbances (12 patients had Lhermitte’s phenomenon) and found that lidocaine abolished the paroxysmal symptom in ten out of 12 patients, thus supporting the ectopically generated high-frequency discharge.

### Mixed pains

#### Painful tonic spasms

This type of pain is specific to MS. Painful tonic spasms are unilateral or bilateral, stereotyped, involuntary muscle contractions that last less than 2 min and may manifest several times a day. These spasms usually continue for weeks or months and then disappear. They can be triggered by touch, movement, hyperventilation, or emotions, and are, though seldom, preceded by a “somesthesic aura” [60, 95, 98]. They may start from the face, arm, or leg, and spread to the adjacent part of the body. Their prevalence ranges from 6 to 11 %. Painful tonic spasms are more common in primary and secondary progressive forms of the disease and their presence correlates positively with age, disease duration, and disability [69]. The spasms originate in the central nervous system from hyperactivity in the central motor fibers, caused by lesions in the internal capsule, cerebral peduncle, medulla, or spinal cord [71, 98].

Although no direct evidence exists, the typically spasmodic muscle contraction suggests ectopically generated high-frequency discharges. Hence, the ideal drugs would be frequency-dependent, voltage-gated, sodium-channel blockers. Accordingly, one controlled trial reported the efficacy of intravenous lidocaine and oral mexiletine [89]. Because of simultaneous activation of adjacent motor units as in muscles cramps, the spasmodic muscle contraction induces extreme vasculature compression, ischemia, and thus activates the muscle nociceptors sensitive to ischemia, eventually giving rise to nociceptive pain (*ischemic muscle pain*) [63]. As soon as the spasm ends the blood flow returns to normal and pain quickly recedes. Cannabinoids, theoretically effective on both neuropathic and nociceptive components, are indeed efficacious, and are more efficacious in painful tonic spasms than the purely neuropathic pains in patients with MS [7, 86].

#### Spasticity pain

Spasticity is an increased tonic stretch reflex, related to Ia presynaptic disinhibition due to corticospinal system damage. It affects about 50–60 % of patients with MS and is often painful [71, 103]. Some movements may focally exacerbate spasticity. These exacerbations, often involving the thigh adductors, are sometimes called contractures or painful spasms, thus engendering confusion with the painful tonic spasms described above. Whereas a clear distinction—clinical and pathophysiological—between painful tonic spasms and the focal exacerbations of spasticity is widely accepted, our review found that trials on spasticity often fail to make the distinction clear. Because most patients with spasticity report pain independently from the occurrence of exacerbations, pain probably reflects the prolonged, abnormal muscle contraction. The responsible mechanism differs from that for painful tonic spasms, because the reflex motoneuronal activation prevents simultaneous contraction of adjacent motor units, so that ischemia is unlikely to develop. As happens when untrained muscles undergo prolonged exercise, muscle receptors are probably activated by the excessive muscular work. This kind of muscle pain (*mechanical muscle pain*) [63] is typical of eccentric contractions such as those in spastic lower-limb extensors when the patient tries to flex the knee during walking. Lengthening a contracted muscle causes structural damage and disruption of even a few muscle fibers releases abundant substances that may directly or indirectly through biochemical changes excite the muscle nociceptors [66]. Although some investigators proposed that the usual algesic substances, such as bradykinin, serotonin, and prostaglandin E2, activate muscle receptors in spasticity [115], mechanical muscle pain seems not to respond to non-steroidal anti-inflammatory drugs [63].

Although pain eventually arises from peripheral nociceptors, evidence underlining that CNS has a primary role comes from the response to treatment, showing that treatments known to relieve spasticity also relieve spasticity-related pain. Several controlled trials found that oral and intrathecal baclofen effectively relieve spasticity and spasticity-related pain [37, 61, 90], as does gabapentin [21, 65]. As expected, cannabinoids, substances that act both on nociceptive and neuropathic pain, proved particularly effective [116].

### Nociceptive pains

#### Nerve trunk pain associated with optic neuritis

Optic neuritis is common in patients with MS. It is the presenting manifestation in about 20 % of patients, and often causes pain. Most studies assessing the prevalence of pain in MS excluded optic neuritis from consideration [71], owing to the lack of widely agreed diagnostic criteria [1]. The only study assessing the prevalence of pain in optic neuritis found a value of 8 % [41]. Pain related to optic neuritis presumably arises from the inflammation of the optic nerve trunk (*nerve trunk pain*) that activates intraneural nociceptors innervated by *nervi nervorum*. The possibility that inflammation directly (ectopically) activates the axons in the *nervi nervorum* seems unlikely, given the dull character of pain.

#### Musculoskeletal pains secondary to postural anomalies and low back pain

Patients with muscle weakness or spasticity are prone to postural anomalies. Abnormal posture in turn perturbs body weight distribution, entails excessive work for some muscles, and induces excessive stress on ligaments and joints. Even reduced mobility alone can lead to osteoporosis, reduce elasticity in tendons and ligaments, and cause joint ankylosis. Among other deleterious consequences, the nociceptive input facilitates homotopical spinal vasoconstrictor neurons, thus increasing pain [28, 50]. This vicious circle must be counteracted from the very beginning, with anti-inflammatory agents and most of all with intensive postural physiotherapy. Our review found no studies estimating the prevalence of musculoskeletal pains (other than low back pain) in MS.

Multiple sclerosis particularly predisposes patients to low back pain. Epidemiological studies reported a prevalence of low back pain in patients with MS ranging from 10 to 16 % [71, 97]. To what extent MS-dependent pathophysiological mechanisms contribute to low back pain remains unclear. Current evidence predominantly attributes low back pain not to central inflammatory processes but to the mechanisms we have already described for musculoskeletal pain in general [69]. Given the insufficient understanding of the pain mechanisms associated with mechanical back pain in general, it is virtually impossible to estimate the importance of mechanisms directly related to MS in patients complaining of low back pain.

#### Headache

All headaches, whether mediated by meningeal, arterial, muscular, or other craniofacial tissue receptors, are nociceptive pains. Whatever its nature, an association seems to exist between headache and MS. Most studies agree that the prevalence of headache is significantly higher in patients with MS patients than in the general population [71]. The reported frequencies vary widely, from 13 to 64 % [52, 71]. In their systematic review, O’Connor and colleagues [71] found three studies specifically assessing the prevalence of headache in MS: out of a total of 269 patients with MS, 86 had a primary form of headache (51 %). Searching the literature published thereafter for studies that focused on assessing the prevalence of headache, provided separate data for migraine and tension-type headache, and had a sample population of at least 100 patients, we found five articles [52, 68, 82, 109, 110]. Out of a total of 1,136 MS patients, 669 had a primary headache (59 %), i.e., a frequency similar to that reported in earlier studies. Interestingly, 381 had migraine with or without aura (34 %) and 242 had tension-type headache (21 %). Whereas the frequency of migraine was significantly higher in patients with MS than in healthy controls, that of tension-type headache was not. Comparing the prevalence of headache in MS with that in the general population in Europe yields even more striking results. The prevalence of migraine is three times higher in patients with MS than in the general population, 34 versus 10 %, as estimated by the European Brain Council [72], whereas the prevalence of tension-type headache is similar, 21 versus 20–34 %, as estimated by epidemiological studies in European Countries [58, 79, 82, 112]. Although some investigators refute MS as a risk factor for headache, the high reported migraine frequency in MS cannot be explained with a mere coexistence of two diseases.

Either MS and migraine share some predisposing factor, or the MS lesions directly induce migraine pain. Several reports have documented that migraine attacks may manifest during symptom exacerbation and may even herald the onset of relapse in MS [71]. Using MRI, a study compared the frequency of lesions in the midbrain (periaqueductal grey, substantia nigra, or red nucleus) in 58 patients with migraine (selected for having supratentorial infarctions), 42 patients with MS without migraine, and 37 patients with MS and migraine: the frequency increased from migraine (23 %), to MS without migraine (32 %), to MS with migraine (52 %) [104]. In a survey of 277 MS patients, 95 having migraine (34 %), patients with a plaque within the midbrain/periaqueductal grey matter (an important area in the antinociceptive control system) as demonstrated by MRI, had a four-fold higher increase in migraine than those without a lesion in that area [29]. Hence, demyelinating lesions in the midbrain, and periaqueductal grey in particular, i.e., areas that are supposedly involved in the pathophysiology of migraine [114], are associated with the development of migraine in MS.

#### Treatment-induced pains

Treatment for MS can also induce secondary nociceptive pains [71]. Interferon beta induces a flu-like syndrome in most patients with MS, some of whom report myalgias that may persist for months. Again interferon beta (but not glatiramer acetate or natalizumab) reportedly increases the frequency and severity of headache. Pain at the injection site for glatiramer acetate is a frequent complaint. Chronic corticosteroid use can cause osteoporosis and secondary pains [3, 71, 77, 80, 85, 111].

### Other types of pain

A number of other types of pain, although far less frequently, have been reported. Central pains affecting the face or trunk, rather than the extremities, resemble the *ongoing extremity pain* and therefore probably arise through similar mechanisms: when somatosensory testing discloses a deficit in thermal-pain sensitivity in the same territory, pain should be attributed to a lesion in the spinothalamic system. Other types of pain include visceral pain and painful entrapment neuropathies secondary to motor disorders; more research is needed before drawing conclusions on both.

## Conclusions

Patients with MS may present with a wide variety of symptoms. This multiform presentation holds true also for pain. We can think of no other disease that can result in so many different types of pain. This review emphasizes how important it is to characterize the type of pain properly in patients with MS. Many epidemiological studies and treatment trials we reviewed reported poor outcomes precisely because they neglected to categorize pain adequately. This drawback impedes a mechanism-based approach.

This review nevertheless attempts a scheme of classification. Admittedly, a great deal of the reasoning is speculative, again because we lack sufficient information on specific types of pain. Future research needs to dig into the mechanisms underlying the various MS-related pains so that treatment can follow a mechanism-based approach.

## Appendix

Systematic search for randomized controlled trials (RCT) assessing the efficacy of medical treatment of pain in multiple sclerosis (MS).

### Search strategy

We searched the PubMed expanded database from inception date to April 2012 using

- the search string: “multiple sclerosis AND (pain OR painful OR analgesic OR antinociceptive)”,
- the limits: “randomized controlled trial” AND “title/abstract”.

Inclusion criteria: placebo-controlled, double-blind RCTs assessing the efficacy of pain-relieving (non disease-modifying) drugs, in patients with multiple sclerosis.

Exclusion criteria: trials in multiple diseases without the possibility of extrapolating the results relative to the patients with multiple sclerosis, trials in less than ten patients with MS, acute or short-lasting treatments.

This search yielded 62 articles. Because one was a duplicate [1], we screened the abstract of 61 articles. Sixteen articles were excluded after reading the abstract because completely not pertinent [2–17]; 13 because assessed non-pharmacological treatments [18–30], six because assessed disease-modifying drugs [31–36], three because assessed treatment-induced pain [37–39], one because assessed experimental pain [40], five because of a small sample size or because the trial assessed multiple diseases without the possibility of extrapolating the results in MS patients, [41–45] four because they were not double-blind or were not placebo-controlled [46–49], and one because the duration of treatment was only 2 days [50]. The remaining 12 studies were selected for analysis [51–62] (see Prisma Chart and List of references).

### References

#### Duplicate

1. Svendsen KB, Jensen TS, Bach FW (2005) Effect of the synthetic cannabinoid dronabinol on central pain in patients with multiple sclerosis—secondary publication. Ugeskr Laeger 167:2772–2774

#### Not pertinent

2. Beck RW, Trobe JD, Moke PS, Gal RL, et al. (2003) Optic Neuritis Study Group. High- and low-risk profiles for the development of multiple sclerosis within 10 years after optic neuritis: experience of the optic neuritis treatment trial. Arch Ophthalmol 121:944–949
3. Bever CT Jr, Anderson PA, Leslie J, Panitch HS, Dhib-Jalbut S, Khan OA, Milo R, Hebel JR, Conway KL, Katz E, Johnson KP (1996) Treatment with oral 3,4 diaminopyridine improves leg strength in multiple sclerosis patients: results of a randomized, double-blind, placebo-controlled, crossover trial. Neurology 47:1457–1462.
4. de Sèze M, Gallien P, Denys P, Labat JJ, Serment G, Grise P, Salle JY, Blazejewski S, Hazane C, Moore N, Joseph PA (2006) Intravesical glucidic capsaicin versus glucidic solvent in neurogenic detrusor overactivity: a double blind controlled randomized study. Neurourol Urodyn 25:752–757.
5. de Sèze M, Wiart L, Joseph PA, Dosque JP, Mazaux JM, Barat M (1998) Capsaicin and neurogenic detrusor hyperreflexia: a double-blind placebo-controlled study in 20 patients with spinal cord lesions. Neurourol Urodyn 17:513–523.
6. Göbel H, Schmid J, Heinze A, Pergande G (1999) Reduction of spastic increased muscle tone in multiple sclerosis by the nonopioid analgesic flupirtine. Schmerz 13:324–331.
7. Hughes RB, Robinson-Whelen S, Taylor HB, Hall JW (2006) Stress self-management: an intervention for women with physical disabilities. Womens Health Issues 16:389–399.
8. Kim JH, Rivas DA, Shenot PJ, Green B, Kennelly M, Erickson JR, O’Leary M, Yoshimura N, Chancellor MB (2003) Intravesical resiniferatoxin for refractory detrusor hyperreflexia: a multicenter, blinded, randomized, placebo-controlled trial. J Spinal Cord Med 26:358–363.
9. Kurzthaler I, Bodner T, Kemmler G, Entner T, Wissel J, Berger T, Fleischhacker WW (2005) The effect of nabilone on neuropsychological functions related to driving ability: an extended case series. Hum Psychopharmacol 20:291–293.
10. Li R, Louie MK, Lee HJ, Osann K, Pick DL, Santos R, McDougall EM, Clayman RV (2011) Prospective randomized trial of three different methods of nephrostomy tract closure after percutaneous nephrolithotripsy. BJU Int 107:1660–1665.
11. Niu YJ, Zang YJ, Chen XG, Li W, Zhu XD, Liu Y, Wei ZY, Zhang ZT, Wang Y, Shen ZY (2006) The study of the different medicating ways and the formula for intravenous loading dosage of hepatitis B immunoglobulin in liver transplantation. Zhonghua Wai Ke Za Zhi 44:1444–1447.
12. Owens JE, Menard M (2011) The quantification of placebo effects within a general model of health care outcomes. J Altern Complement Med 17:817–821.
13. Rajatanavin R, Chailurkit L, Chiemchanya S (1994) The efficacy of percutaneous tetracycline instillation for sclerosis of recurrent thyroid cysts: a multivariate analysis. J Endocrinol Invest 17:123–125.
14. Stenager E, Knudsen L, Jensen K (1995) Acute and chronic pain syndromes in multiple sclerosis. A 5-year follow-up study. Ital J Neurol Sci 16(9):629–632.
15. Stuifbergen AK, Becker H, Blozis S, Timmerman G, Kullberg V (2003) A randomized clinical trial of a wellness intervention for women with multiple sclerosis. Arch Phys Med Rehabil 84:467–476.
16. Optic Neuritis Study Group (1997) The 5-year risk of MS after optic neuritis. Experience of the optic neuritis treatment trial. Neurology 49:1404–1413.
17. Van Diemen HA, Polman CH, Koetsier JC, Van Loenen AC, Nauta JJ, Bertelsmann FW (1993) 4-Aminopyridine in patients with multiple sclerosis: dosage and serum level related to efficacy and safety. Clin Neuropharmacol 16:195–204.

#### Non-pharmacological treatment

18. Al-Smadi J, Warke K, Wilson I, Cramp AF, Noble G, Walsh DM, Lowe-Strong AS (2003) A pilot investigation of the hypoalgesic effects of transcutaneous electrical nerve stimulation upon low back pain in people with multiple sclerosis. Clin Rehabil 17:742–749.
19. Collett J, Dawes H, Meaney A, Sackley C, Barker K, Wade D, Izardi H, Bateman J, Duda J, Buckingham E (2011) Exercise for multiple sclerosis: a single-blind randomized trial comparing three exercise intensities. Mult Scler 17:594–603.
20. Donnellan CP, Shanley J (2008) Comparison of the effect of two types of acupuncture on quality of life in secondary progressive multiple sclerosis: a preliminary single-blind randomized controlled trial. Clin Rehabil 22:195–205.
21. Higginson IJ, Costantini M, Silber E, Burman R, Edmonds P (2011) Evaluation of a new model of short-term palliative care for people severely affected with multiple sclerosis: a randomised fast-track trial to test timing of referral and how long the effect is maintained. Postgrad Med J 87:769–775.
22. Hughes CM, Smyth S, Lowe-Strong AS (2009) Reflexology for the treatment of pain in people with multiple sclerosis: a double-blind randomised sham-controlled clinical trial. Mult Scler 15:1329–1338.
23. Jensen MP, Ehde DM, Gertz KJ, Stoelb BL, Dillworth TM, Hirsh AT, Molton IR, Kraft GH (2011) Effects of self-hypnosis training and cognitive restructuring on daily pain intensity and catastrophizing in individuals with multiple sclerosis and chronic pain. Int J Clin Exp Hypn 59:45–63.
24. Miller L, Mattison P, Paul L, Wood L (2007) The effects of transcutaneous electrical nerve stimulation (TENS) on spasticity in multiple sclerosis. Mult Scler 13:527–533.
25. Mori F, Codecà C, Kusayanagi H, Monteleone F, Buttari F, Fiore S, Bernardi G, Koch G, Centonze D (2010) Effects of anodal transcranial direct current stimulation on chronic neuropathic pain in patients with multiple sclerosis. J Pain 11:436–442.
26. Nilsagård Y, Denison E, Gunnarsson LG (2006) Evaluation of a single session with cooling garment for persons with multiple sclerosis--a randomized trial. Disabil Rehabil Assist Technol 1:225–233.
27. Patti F, Ciancio MR, Reggio E, Lopes R, Palermo F, Cacopardo M, Reggio A (2002) The impact of outpatient rehabilitation on quality of life in multiple sclerosis. J Neurol 249:1027–1033.
28. Pozzilli C, Brunetti M, Amicosante AM, Gasperini C, Ristori G, Palmisano L, Battaglia M (2002) Home based management in multiple sclerosis: results of a randomised controlled trial. J Neurol Neurosurg Psychiatry 73:250–255.
29. Storr LK, Sørensen PS, Ravnborg M (2006) The efficacy of multidisciplinary rehabilitation in stable multiple sclerosis patients. Mult Scler 12:235–242.
30. Warke K, Al-Smadi J, Baxter D, Walsh DM, Lowe-Strong AS (2006) Efficacy of transcutaneous electrical nerve stimulation (tens) for chronic low back pain in a multiple sclerosis population: a randomized, placebo-controlled clinical trial. Clin J Pain 22:812–819.

#### Disease-modifying drugs

31. Andersen O, Lycke J, Tollesson PO, Svenningsson A, Runmarker B, Linde AS, Aström M, Gjörstrup P, Ekholm S (1996) Linomide reduces the rate of active lesions in relapsing-remitting multiple sclerosis. Neurology 47:895–900.
32. Anderson G, Meyer D, Herrman CE, Sheppard C, Murray R, Fox EJ, Mathena J, Conner J, Buck PO (2010) Tolerability and safety of novel half milliliter formulation of glatiramer acetate for subcutaneous injection: an open-label, multicenter, randomized comparative study. J Neurol 257:1917–1923.
33. Hurwitz BJ, Jeffery D, Arnason B, Bigley K, Coyle P, Goodin D, Kaba S, Kirzinger S, Lynch S, Mandler R, Mikol D, Rammohan K, Sater R, Sriram S, Thrower B, Boateng F, Jakobs P, Wash MB, Bogumil T (2008) Tolerability and safety profile of 12- to 28-week treatment with interferon beta-1b 250 and 500 microg QOD in patients with relapsing-remitting multiple sclerosis: a multicenter, randomized, double-blind, parallel-group pilot study. Clin Ther 30:1102–1112.
34. Kappos L, Gold R, Miller DH, Macmanus DG, Havrdova E, Limmroth V, Polman CH, Schmierer K, Yousry TA, Yang M, Eraksoy M, Meluzinova E, Rektor I, Dawson KT, Sandrock AW, O’Neill GN, BG-12 Phase IIb Study Investigators (2008) Efficacy and safety of oral fumarate in patients with relapsing-remitting multiple sclerosis: a multicentre, randomised, double-blind, placebo-controlled phase IIb study. Lancet 372:1463–1472.
35. Simsarian JP, Saunders C, Smith DM (2011) Five-day regimen of intramuscular or subcutaneous self-administered adrenocorticotropic hormone gel for acute exacerbations of multiple sclerosis: a prospective, randomized, open-label pilot trial. Drug Des Devel Ther 5:381–389.
36. The Lenercept Multiple Sclerosis Study Group and The University of British Columbia MS/MRI Analysis Group (1999) TNF neutralization in MS: results of a randomized, placebo-controlled multicenter study. Neurology 53:457–465.

#### Treatment-induced pain

37. Brochet B, Lemaire G, Beddiaf A; et l’Epicure Study Group (2006) Reduction of injection site reactions in multiple sclerosis (MS) patients newly started on interferon beta 1b therapy with two different devices. Rev Neurol (Paris) 162:735–740.
38. Jolly H, Simpson K, Bishop B, Hunter H, Newell C, Denney D, Oleen-Burkey M (2008) Impact of warm compresses on local injection-site reactions with self-administered glatiramer acetate. J Neurosci Nurs 40:232–239.
39. Pardo G, Boutwell C, Conner J, Denney D, Oleen-Burkey M (2010) Effect of oral antihistamine on local injection site reactions with self-administered glatiramer acetate. J Neurosci Nurs 42:40–46.

#### Experimental pain

40. Conte A, Bettolo CM, Onesti E, Frasca V, Iacovelli E, Gilio F, Giacomelli E, Gabriele M, Aragona M, Tomassini V, Pantano P, Pozzilli C, Inghilleri M (2009) Cannabinoid-induced effects on the nociceptive system: a neurophysiological study in patients with secondary progressive multiple sclerosis. Eur J Pain 13:472–477.

#### Insufficient sample (or multiple diseases with results in ms impossible to extrapolate)

41. Deutsch SI, Rosse RB, Connor JM, Burket JA, Murphy ME, Fox FJ (2008) Current status of cannabis treatment of multiple sclerosis with an illustrative case presentation of a patient with MS, complex vocal tics, paroxysmal dystonia, and marijuana dependence treated with dronabinol. CNS Spectr 13:393–403.
42. Herman RM, D’Luzansky SC, Ippolito R (1992) Intrathecal baclofen suppresses central pain in patients with spinal lesions. A pilot study. Clin J Pain 8:338–345.
43. Silver M, Blum D, Grainger J, Hammer AE, Quessy S (2007) Double-blind, placebo-controlled trial of lamotrigine in combination with other medications for neuropathic pain. J Pain Symptom Manage 34:446–54.
44. Wade DT, Robson P, House H, Makela P, Aram J (2003) A preliminary controlled study to determine whether whole-plant cannabis extracts can improve intractable neurogenic symptoms. Clin Rehabil 17:21–9.
45. Wissel J, Haydn T, Müller J, Brenneis C, Berger T, Poewe W, Schelosky LD (2006) Low dose treatment with the synthetic cannabinoid Nabilone significantly reduces spasticity-related pain: a double-blind placebo-controlled cross-over trial. J Neurol 253:1337–1341.

#### Insufficient control (no placebo or not double-blind)

46. Chitsaz A, Janghorbani M, Shaygannejad V, Ashtari F, Heshmatipour M, Freeman J (2009) Sensory complaints of the upper extremities in multiple sclerosis: relative efficacy of nortriptyline and transcutaneous electrical nerve stimulation. Clin J Pain 25:281–285.
47. Leuschen MP, Filipi M, Healey K (2004) A randomized open label study of pain medications (naproxen, acetaminophen and ibuprofen) for controlling side effects during initiation of IFN beta-1a therapy and during its ongoing use for relapsing-remitting multiple sclerosis. Mult Scler 10:636–642.
48. Rog DJ, Nurmikko TJ, Young CA (2007) Oromucosal delta9-tetrahydrocannabinol/cannabidiol for neuropathic pain associated with multiple sclerosis: an uncontrolled, open-label, 2-year extension trial. Clin Ther 29:2068–2079.
49. Wade DT, Makela PM, House H, Bateman C, Robson P (2006) Long-term use of a cannabis-based medicine in the treatment of spasticity and other symptoms in multiple sclerosis. Mult Scler 12:639–645.

#### Insufficient duration of treatment

50. Mueller ME, Gruenthal M, Olson WL, Olson WH (1997) Gabapentin for relief of upper motor neuron symptoms in multiple sclerosis. Arch Phys Med Rehabil 78:521–524.

#### Selected

51. Breuer B, Pappagallo M, Knotkova H, Guleyupoglu N, Wallenstein S, Portenoy RK (2007) A randomized, double-blind, placebo-controlled, two-period, crossover, pilot trial of lamotrigine in patients with central pain due to multiple sclerosis. Clin Ther 29:2022–2030.
52. Cree BA, Kornyeyeva E, Goodin DS (2010) Pilot trial of low-dose naltrexone and quality of life in multiple sclerosis. Ann Neurol 68:145–150.
53. Cutter NC, Scott DD, Johnson JC, Whiteneck G (2000) Gabapentin effect on spasticity in multiple sclerosis: a placebo-controlled, randomized trial. Arch Phys Med Rehabil 81:164–169.
54. Hyman N, Barnes M, Bhakta B, Cozens A, Bakheit M, Kreczy-Kleedorfer B, Poewe W, Wissel J, Bain P, Glickman S, Sayer A, Richardson A, Dott C (2000) Botulinum toxin (Dysport) treatment of hip adductor spasticity in multiple sclerosis: a prospective, randomised, double blind, placebo controlled, dose ranging study. J Neurol Neurosurg Psychiatry 68:707–712.
55. Loder C, Allawi J, Horrobin DF (2002) Treatment of multiple sclerosis with lofepramine, l-phenylalanine and vitamin B(12): mechanism of action and clinical importance: roles of the locus coeruleus and central noradrenergic systems. Med Hypotheses 59:594–602.
56. Panitch HS, Thisted RA, Smith RA, Wynn DR, Wymer JP, Achiron A, Vollmer TL, Mandler RN, Dietrich DW, Fletcher M, Pope LE, Berg JE, Miller A; Psuedobulbar Affect in Multiple Sclerosis Study Group (2006) Randomized, controlled trial of dextromethorphan/quinidine for pseudobulbar affect in multiple sclerosis. Ann Neurol 59:780–787.
57. Rog DJ, Nurmikko TJ, Friede T, Young CA (2005) Randomized, controlled trial of cannabis-based medicine in central pain in multiple sclerosis. Neurology 65:812–819.
58. Rossi S, Mataluni G, Codecà C, Fiore S, Buttari F, Musella A, Castelli M, Bernardi G, Centonze D (2009) Effects of levetiracetam on chronic pain in multiple sclerosis: results of a pilot, randomized, placebo-controlled study. Eur J Neurol 16:360–366.
59. Sharafaddinzadeh N, Moghtaderi A, Kashipazha D, Majdinasab N, Shalbafan B (2010) The effect of low-dose naltrexone on quality of life of patients with multiple sclerosis: a randomized placebo-controlled trial. Mult Scler 16:964–969.
60. Svendsen KB, Jensen TS, Bach FW (2004) Does the cannabinoid dronabinol reduce central pain in multiple sclerosis? Randomised double blind placebo controlled crossover trial. BMJ 329:253.
61. Wade DT, Makela P, Robson P, House H, Bateman C (2004) Do cannabis-based medicinal extracts have general or specific effects on symptoms in multiple sclerosis? A double-blind, randomized, placebo-controlled study on 160 patients. Mult Scler 10:434–441.
62. Zajicek J, Fox P, Sanders H, Wright D, Vickery J, Nunn A, Thompson A; UK MS Research Group (2003) Cannabinoids for treatment of spasticity and other symptoms related to multiple sclerosis (CAMS study): multicentre randomised placebo-controlled trial. Lancet 362:1517–1526.

## References

[1] Agostoni E, Frigerio R, Protti A (2005). Controversies in optic neuritis pain diagnosis. Neurol Sci.

[2] Al-Araji AH, Oger J (2005). Reappraisal of Lhermitte’s sign in multiple sclerosis. Mult Scler.

[3] Anderson G, Meyer D, Herrman CE (2010). Tolerability and safety of novel half milliliter formulation of glatiramer acetate for subcutaneous injection: an open-label, multicenter, randomized comparative study. J Neurol.

[4] Archibald CJ, McGrath PJ, Ritvo PG (1994). Pain prevalence, severity and impact in a clinic sample of multiple sclerosis patients. Pain.

[5] Athanasiou TC, Patel NK, Renowden SA, Coakham HB (2005). Some patients with multiple sclerosis have neurovascular compression causing their trigeminal neuralgia and can be treated effectively with MVD: report of five cases. Br J Neurosurg.

[6] Attal N, Bouhassira D, Baron R, Dostrovsky J, Dworkin RH, Finnerup N, Gourlay G, Haanpaa M, Raja S, Rice AS, Simpson D, Treede RD (2011). Assessing symptom profiles in neuropathic pain clinical trials: can it improve outcome?. Eur J Pain.

[7] Attal N, Cruccu G, Baron R, Haanpää M, Hansson P, Jensen TS, Nurmikko T (2010). EFNS guidelines on the pharmacological treatment of neuropathic pain: 2010 revision. Eur J Neurol.

[8] Baron R, Binder A (2004). How neuropathic is sciatica? The mixed pain concept. Orthopade.

[9] Baron R, Tölle TR, Gockel U, Brosz M, Freynhagen R (2009). A cross-sectional cohort survey in 2100 patients with painful diabetic neuropathy and postherpetic neuralgia: differences in demographic data and sensory symptoms. Pain.

[10] Beiske AG, Pedersen ED, Czujko B, Myhr KM (2004). Pain and sensory complaints in multiple sclerosis. Eur J Neurol.

[11] Boneschi FM, Colombo B, Annovazzi P (2008). Lifetime and actual prevalence of pain and headache in multiple sclerosis. Mult Scler.

[12] Broggi G, Ferroli P, Franzini A, Nazzi V, Farina L, La Mantia L (2004). Operative findings and outcomes of microvascular decompression for trigeminal neuralgia in 35 patients affected by multiple sclerosis. Neurosurgery.

[13] Breuer B, Pappagallo M, Knotkova H, Guleyupoglu N, Wallenstein S, Portenoy RK (2007). A randomized, double-blind, placebo-controlled, two-period, crossover, pilot trial of lamotrigine in patients with central pain due to multiple sclerosis. Clin Ther.

[14] Cervero F, Laird JMA (1991). One pain or many pains? A new look at pain mechanisms. News Physiol Sci.

[15] Cree BA, Kornyeyeva E, Goodin DS (2010). Pilot trial of low-dose naltrexone and quality of life in multiple sclerosis. Ann Neurol.

[16] Cruccu G, Biasiotta A, Di Rezze S, Fiorelli M, Galeotti F, Innocenti P, Mameli S, Millefiorini E, Truini A (2009). Trigeminal neuralgia and pain related to multiple sclerosis. Pain.

[17] Cruccu G, Gronseth G, Alksne J, Argoff C, Brainin M, Burchiel K (2008). AAN-EFNS guidelines on trigeminal neuralgia management. Eur J Neurol.

[18] Cruccu G, Iannetti GD, Marx JJ, Thoemke F, Truini A, Fitzek S (2005). Brainstem reflex circuits revisited. Brain.

[19] Cruccu G, Sommer C, Anand P, Attal N, Baron R, Garcia-Larrea L, Haanpaa M, Jensen TS, Serra J, Treede RD (2010). EFNS guidelines on neuropathic pain assessment: revised 2009. Eur J Neurol.

[20] Cruccu G, Truini A (2009). Sensory profiles: a new strategy for selecting patients in treatment trials for neuropathic pain. Pain.

[21] Cutter NC, Scott DD, Johnson JC, Whiteneck G (2000). Gabapentin effect on spasticity in multiple sclerosis: a placebo-controlled, randomized trial. Arch Phys Med Rehabil.

[22] Daousi C, Benbow SJ, Woodward A, MacFarlane IA (2006). The natural history of chronic painful peripheral neuropathy in a community diabetes population. Diabet Med.

[23] Davies M, Brophy S, Williams R, Taylor A (2006). The prevalence, severity, and impact of painful diabetic peripheral neuropathy in type 2 diabetes. Diabetes Care.

[24] Diatchenko L, Nackley AG, Slade GD, Fillingim RB, Maixner W (2006). Idiopathic pain disorders–pathways of vulnerability. Pain.

[25] Eldridge PR, Sinha AK, Javadpour M, Littlechild P, Varma TR (2003). Microvascular decompression for trigeminal neuralgia in patients with multiple sclerosis. Stereotact Funct Neurosurg.

[26] Finnerup NB, Jensen TS (2005). Mechanisms of disease: mechanism-based classification of neuropathic pain—a critical analysis. Nat Clin Pract Neurol.

[27] Freynhagen R, Baron R (2009). The evaluation of neuropathic components in low back pain. Curr Pain Headache Rep.

[28] Ge HY, Fernandez-de-las-Penas C, Arendt-Nielsen L (2006). Sympathetic facilitation of hyperalgesia evoked from myofascial tender and trigger points in patients with unilateral shoulder pain. Clin Neurophysiol.

[29] Gee JR, Chang J, Dublin AB, Vijayan N (2005). The association of brainstem lesions with migraine-like headache: an imaging study of multiple sclerosis. Headache.

[30] Goldenberg DL (2010). Pain/depression dyad: a key to a better understanding and treatment of functional somatic syndromes. Am J Med.

[31] Gronseth G, Cruccu G, Alksne J, Argoff C, Brainin M, Burchiel K (2008). Practice Parameter: the diagnostic evaluation and treatment of trigeminal neuralgia (an evidence-based review). Report of the Quality Standards Subcommittee of the American Academy of Neurology and the European Federation of Neurological Societies. Neurology.

[32] Gutrecht JA, Zamani AA, Slagado ED (1993). Anatomic–radiologic basis of Lhermitte’s sign in multiple sclerosis. Arch Neurol.

[33] Haanpaa M, Attal N, Backonja M (2011). NeuPSIG guidelines on neuropathic pain assessment. Pain.

[34] Hadjimichael O, Kerns RD, Rizzo MA (2007). Persistent pain and uncomfortable sensations in persons with multiple sclerosis. Pain.

[35] Headache Classification Subcommittee of the International Headache Society (2004). The international classification of headache disorders, 2nd edn. Cephalalgia.

[36] Henningsen P, Löwe B (2006) Depression, pain, and somatoform disorders. Curr Opin Psychiatry 19:19–24 10.1097/01.yco.0000189880.11059.8d 16612174

[37] Herman RM, D’Luzansky SC, Ippolito R (1992). Intrathecal baclofen suppresses central pain in patients with spinal lesions. A pilot study. Clin J Pain.

[38] Hooge JP, Redekop WK (1995). Trigeminal neuralgia in multiple sclerosis. Neurology.

[39] Hyman N, Barnes M, Bhakta B (2000). Botulinum toxin (Dysport) treatment of hip adductor spasticity in multiple sclerosis: a prospective, randomised, double blind, placebo controlled, dose ranging study. J Neurol Neurosurg Psychiatry.

[40] Merskey H, Bogduk N, IASP Task Force on Taxonomy (1994). Classification of chronic pain. 2nd edn.

[41] Indaco A, Iachetta C, Nappi C, Socci L, Carrieri PB (1994). Chronic and acute pain syndromes in patients with multiple sclerosis. Acta Neurol (Napoli).

[42] Inui K, Wang X, Qiu Y, Nguyen BT, Ojima S, Tamura Y, Nakata H, Wasaka T, Tran TD, Kakigi R (2003). Pain processing within the primary somatosensory cortex in humans. Eur J Neurosci.

[43] Jensen TS, Rasmussen P, Reske-Nielsen E (1982). Association of trigeminal neuralgia with multiple sclerosis: clinical and pathological features. Acta Neurol Scand.

[44] Kalia LV, O’Connor PW (2005). Severity of chronic pain and its relationship to quality of life in multiple sclerosis. Mult Scler.

[45] Kanchandani R, Howe JG (1982) Lhermitte’s sign in multiple sclerosis: a clinical survey and review of the literature. J Neurol Neurosurg Psychiatry 45:308–212 10.1136/jnnp.45.4.308 PMC491365 7077340

[46] Kanda M, Nagamine T, Ikeda A, Ohara S, Kunieda T, Fujiwara N, Yazawa S, Sawamoto N, Matsumoto R, Taki W, Shibasaki H (2000). Primary somatosensory cortex is actively involved in pain processing in human. Brain Res.

[47] Katona C, Peveler R, Dowrick C, Wessely S, Feinmann C, Gask L, Lloyd H, Williams AC, Wager E (2005). Pain symptoms in depression: definition and clinical significance. Clin Med.

[48] Katusic S, Williams DB, Beard CM, Bergstralh EJ, Kurland LT (1991). Epidemiology and clinical features of idiopathic trigeminal neuralgia and glossopharyngeal neuralgia: similarities and differences, Rochester, Minnesota, 1945–1984. Neuroepidemiology.

[49] Kerba M, Wu JS, Duan Q, Hagen NA, Bennett MI (2010). Neuropathic pain features in patients with bone metastases referred for palliative radiotherapy. J Clin Oncol.

[50] Kimura Y, Ge HY, Zhang Y, Kimura M, Sumikura H, Arendt-Nielsen L (2009). Evaluation of sympathetic vasoconstrictor response following nociceptive stimulation of latent myofascial trigger points in humans. Acta Physiol (Oxf).

[51] Kindler LL, Bennett RM, Jones KD (2011). Central sensitivity syndromes: mounting pathophysiologic evidence to link fibromyalgia with other common chronic pain disorders. Pain Manag Nurs.

[52] Kister I, Caminero AB, Monteith TS, Soliman A, Bacon TE, Bacon JH, Kalina JT, Inglese M, Herbert J, Lipton RB (2010). Migraine is comorbid with multiple sclerosis and associated with a more symptomatic MS course. J Headache Pain.

[53] Lépine JP, Briley M (2004). The epidemiology of pain in depression. Hum Psychopharmacol.

[54] Loder C, Allawi J, Horrobin DF (2002). Treatment of multiple sclerosis with lofepramine, l-phenylalanine and vitamin B(12): mechanism of action and clinical importance: roles of the locus coeruleus and central noradrenergic systems. Med Hypotheses.

[55] Love S, Coakham HB (2001). Trigeminal neuralgia: pathology and pathogenesis. Brain.

[56] Love S, Gradidge T, Coakham HB (2001). Trigeminal neuralgia due to multiple sclerosis: ultrastructural findings in trigeminal rhizotomy specimens. Neuropathol Appl Neurobiol.

[57] Love S, Hilton DA, Coakham HB (1998). Central demyelination of the Vth nerve root in trigeminal neuralgia associated with vascular compression. Brain Pathol.

[58] Lyngberg AC, Rasmussen BK, Jørgensen T, Jensen R (2005). Has the prevalence of migraine and tension-type headache changed over a 12-year period? A Danish population survey. Eur J Epidemiol.

[59] Maier C, Baron R, Tölle TR (2010). Quantitative sensory testing in the German Research Network on Neuropathic Pain (DFNS): somatosensory abnormalities in 1236 patients with different neuropathic pain syndromes. Pain.

[60] Matthews WB (1975). Paroxysmal symptoms in multiple sclerosis. J Neurol Neurosurg Psychiatry.

[61] Middel B, Kuipers-Upmeijer H, Bouma J (1997). Effect of intrathecal baclofen delivered by an implanted programmable pump on health related quality of life in patients with severe spasticity. J Neurol Neurosurg Psychiatry.

[62] Middlemiss T, Laird BJ, Fallon MT (2011). Mechanisms of cancer-induced bone pain. Clin Oncol (R Coll Radiol).

[63] Mills KR, Newham DJ, Edwards RHT, Wall PD, Melzack R (1989). Muscle pain. Textbook of pain.

[64] Morales Y, Parisi JE, Lucchinetti CF, Freedman MS (2007). The pathology of multiple sclerosis: evidence for eterogeneity. Advances in neurology.

[65] Mueller ME, Gruenthal M, Olson WL, Olson WH (1997). Gabapentin for relief of upper motor neuron symptoms in multiple sclerosis. Arch Phys Med Rehabil.

[66] Newham DJ, McPhail G, Mills KR, Edwards RH (1983). Ultrastructural changes after concentric and eccentric contractions of human muscle. J Neurol Sci.

[67] Nickel JC, Tripp DA, Pontari M, Moldwin R, Mayer R, Carr LK, Doggweiler R, Yang CC, Mishra N, Nordling J (2010). Interstitial cystitis/painful bladder syndrome and associated medical conditions with an emphasis on irritable bowel syndrome, fibromyalgia and chronic fatigue syndrome. J Urol.

[68] Nicoletti A, Patti F, Lo Fermo S, Liberto A, Castiglione A, Laisa P, Garifoli A, La Naia F, Maimone D, Sorbello V, Contrafatto D, Zappia M (2008) Headache and multiple sclerosis: a population-based case-control study in Catania, Sicily. Cephalalgia 28:1163–1169 10.1111/j.1468-2982.2008.01662.x 18727645

[69] Nurmikko TJ, Gupta S, Maclver K (2010). Multiple sclerosis-related central pain disorders. Curr Pain Headache Rep.

[70] Obermann M, Katsarava Z (2009). Update on trigeminal neuralgia. Expert Rev Neurother.

[71] O’Connor AB, Schwid SR, Herrmann DN, Markman JD, Dworkin RH (2008). Pain associated with multiple sclerosis: systematic review and proposed classification. Pain.

[72] Olesen J, Baker MG, Freund T, di Luca M, Mendlewicz J, Ragan I, Westphal M (2006). Consensus document on European brain research. J Neurol Neurosurg Psychiatry.

[73] Österberg A, Boivie J, Thuomas K (2005). Central pain in multiple sclerosis—prevalence and clinical characteristics. Eur J Pain.

[74] Österberg A, Boivie J (2010). Central pain in multiple sclerosis—sensory abnormalities. Eur J Pain.

[75] Panitch HS, Thisted RA, Smith RA, Wynn DR, Wymer JP, Achiron A, Vollmer TL, Mandler RN, Dietrich DW, Fletcher M, Pope LE, Berg JE, Miller A (2006). Psuedobulbar Affect in Multiple Sclerosis Study Group. Randomized, controlled trial of dextromethorphan/quinidine for pseudobulbar affect in multiple sclerosis. Ann Neurol.

[76] Pantano F, Zoccoli A, Iuliani M, Lanzetta G, Vincenzi B, Tonini G, Santini D (2011). New targets, new drugs for metastatic bone pain: a new philosophy. Expert Opin Emerg Drugs.

[77] Patti F, Nicoletti A, Pappalardo A, Castiglione A, Lo Fermo S, Messina S, D’Amico E, Cimino V, Zappia M (2011) Frequency and severity of headache is worsened by Interferon-β therapy in patients with multiple sclerosis. Acta Neurol Scand. doi: 10.1111/j.1600-0404.2011.01532.x 10.1111/j.1600-0404.2011.01532.x 21649611

[78] Periquet MI, Novak V, Collins MP, Nagaraja HN, Erdem S, Nash SM (1999). Painful sensory neuropathy: prospective evaluation using skin biopsy. Neurology.

[79] Pfaffenrath V, Fendrich K, Vennemann M, Meisinger C, Ladwig KH, Evers S, Straube A, Hoffmann W, Berger K (2009). Regional variations in the prevalence of migraine and tension-type headache applying the new IHS criteria: the German DMKG Headache Study. Cephalalgia.

[80] Pollmann W, Erasmus LP, Feneberg W (2002). Interferon beta but not glatiramer acetate therapy aggravates headaches in MS. Neurology.

[81] Pozzilli C, Brunetti M, Amicosante AM, Gasperini C, Ristori G, Palmisano L, Battaglia M (2002). Home based management in multiple sclerosis: results of a randomised controlled trial. J Neurol Neurosurg Psychiatry.

[82] Putzki N, Pfriem A, Limmroth V, Yaldizli O, Tettenborn B, Diener HC, Katsarava Z (2009). Prevalence of migraine, tension-type headache and trigeminal neuralgia in multiple sclerosis. Eur J Neurol.

[83] Quiton RL, Masri R, Thompson SM, Keller A (2010). Abnormal activity of primary somatosensory cortex in central pain syndrome. J Neurophysiol.

[84] Rief W, Zenz M, Schweiger U, Rüddel H, Henningsen P, Nilges P (2008). Redefining (somatoform) pain disorder in ICD-10: a compromise of different interest groups in Germany. Curr Opin Psychiatry.

[85] Río J, Nos C, Bonaventura I, Arroyo R, Genis D, Sureda B, Ara JR, Brieva L, Martín J, Saiz A, Sánchez López F, Prieto JM, Roquer J, Dorado JF, Montalban X (2004) Corticosteroids, ibuprofen, and acetaminophen for IFNbeta-1a flu symptoms in MS: a randomized trial. Neurology 63:525–528 10.1212/01.wnl.0000133206.44931.25 15304586

[86] Rog DJ, Nurmikko TJ, Friede T, Young CA (2005). Randomized, controlled trial of cannabis-based medicine in central pain in multiple sclerosis. Neurology.

[87] Ross E (2001). Moving towards rational pharmacological management of pain with an improved classification system of pain. Expert Opin Pharmacother.

[88] Rossi S, Mataluni G, Codecà C, Fiore S, Buttari F, Musella A, Castelli M, Bernardi G, Centonze D (2009). Effects of levetiracetam on chronic pain in multiple sclerosis: results of a pilot, randomized, placebo-controlled study. Eur J Neurol.

[89] Sakurai M, Kanazawa I (1999). Positive symptoms in multiple sclerosis: their treatment with sodium channel blockers, lidocaine and mexiletine. J Neurol Sci.

[90] Sawa GM, Paty DW (1979). The use of baclofen in treatment of spasticity in multiple sclerosis. Can J Neurol Sci.

[91] Scadding JW, Koltzenburg M. Neuropathic pain (2005) In: McMahon SB, Koltzenburg M (eds) Textbook of pain, 5th edn. Churchill-Livingstone, Edinburgh, pp 973–999

[92] Schaltenbrand G, Wahren W (1977). Atlas for stereotaxy of the human brain.

[93] Sen D, Christie D (2006). Chronic idiopathic pain syndromes. Best Pract Res Clin Rheumatol.

[94] Sharafaddinzadeh N, Moghtaderi A, Kashipazha D, Majdinasab N, Shalbafan B (2010). The effect of low-dose naltrexone on quality of life of patients with multiple sclerosis: a randomized placebo-controlled trial. Mult Scler.

[95] Shibasaki H, Kuroiwa Y (1974). Painful tonic seizures in multiple sclerosis. Arch Neurol.

[96] Smith HS, Barkin RL (2010). Fibromyalgia syndrome: a discussion of the syndrome and pharmacotherapy. Am J Ther.

[97] Solaro C, Brichetto G, Amato MP, Cocco E, Colombo B, D’Aleo G (2004). The prevalence of pain in multiple sclerosis: a multicenter cross-sectional study. Neurology.

[98] Spissu A, Cannas A, Ferrigno P (1999). Anatomic correlates of painful tonic spasms in multiple sclerosis. Mov Disord.

[99] Sullivan M (2004). Exaggerated pain behavior: by what standard?. Clin J Pain.

[100] Svendsen KB, Jensen TS, Bach FW (2004). Does the cannabinoid dronabinol reduce central pain in multiple sclerosis? Randomised double blind placebo controlled crossover trial. BMJ.

[101] Svendsen KB, Jensen TS, Overvad K, Hansen HJ, Koch-Henriksen N, Bach FW (2003). Pain in patients with multiple sclerosis: a population-based study. Arch Neurol.

[102] Tan T, Barry P, Reken S, Baker M, Guideline Development Group (2010) Pharmacological management of neuropathic pain in non-specialist settings: summary of NICE guidance. BMJ 340:c1079 10.1136/bmj.c1079 20335333

[103] Thompson AJ, Toosy AT, Ciccarelli O (2010). Pharmacological management of symptoms in multiple sclerosis: current approaches and future directions. Lancet Neurol.

[104] Tortorella P, Rocca MA, Colombo B, Annovazzi P, Comi G, Filippi M (2006). Assessment of MRI abnormalities of the brainstem from patients with migraine and multiple sclerosis. J Neurol Sci.

[105] Treede RD, Jensen TS, Campbell JN, Cruccu G, Dostrovsky JO, Griffin JW, Hansson P, Hughes R, Nurmikko T, Serra J (2008). Neuropathic pain: redefinition and a grading system for clinical and research purposes. Neurology.

[106] Treede RD, Meyer RA, Raja SN, Campbell JN (1992). Peripheral and central mechanisms of cutaneous hyperalgesia. Prog Neurobiol.

[107] Truini A, Galeotti F, Cruccu G (2011). Treating pain in multiple sclerosis. Expert Opin Pharmacother.

[108] Turk DC, Flor H (1987). Pain greater than pain behaviors: the utility and limitations of the pain behavior construct. Pain.

[109] Vacca G, Marano E, Brescia Morra V, Lanzillo R, De Vito M, Parente E, Orefice G (2007) Multiple sclerosis and headache co-morbidity. A case-control study. Neurol Sci 28:133–135 10.1007/s10072-007-0805-1 17603764

[110] Villani V, Prosperini L, Ciuffoli A, Pizzolato R, Salvetti M, Pozzilli C, Sette G (2008). Primary headache and multiple sclerosis: preliminary results of a prospective study. Neurol Sci.

[111] Villani V, Prosperini L, De Giglio L, Pozzilli C, Salvetti M, Sette G (2012). The impact of interferon beta and natalizumab on comorbid migraine in multiple sclerosis. Headache.

[112] Vuković V, Plavec D, Pavelin S, Janculjak D, Ivanković M, Demarin V (2010). Prevalence of migraine, probable migraine and tension-type headache in the Croatian population. Neuroepidemiology.

[113] Wade DT, Makela P, Robson P, House H, Bateman C (2004). Do cannabis-based medicinal extracts have general or specific effects on symptoms in multiple sclerosis? A double-blind, randomized, placebo-controlled study on 160 patients. Mult Scler.

[114] Welch KM, Nagesh V, Aurora SK, Gelman N (2001). Periaqueductal gray matter dysfunction in migraine: cause or the burden of illness?. Headache.

[115] Wissel J, Müller J, Dressnandt J (2000). Management of spasticity associated pain with botulinum toxin A. J Pain Symptom Manage.

[116] Zajicek J, Fox P, Sanders H, Wright D, Vickery J, Nunn A, Thompson A; UK MS Research Group (2003). Cannabinoids for treatment of spasticity and other symptoms related to multiple sclerosis (CAMS study): multicentre randomised placebo-controlled trial. Lancet.

[117] Zhao J, Pan HL, Li TT, Zhang YQ, Wei JY, Zhao ZQ (2010). The sensitization of peripheral C-fibers to lysophosphatidic acid in bone cancer pain. Life Sci.

